# Supplementary material for: East Antarctic warming forced by ice loss during the Last Interglacial
Source: Nat Commun. 2024 Feb 3;15:1026. doi: 10.1038/s41467-024-45501-x (PMC10838265; doi:10.1038/s41467-024-45501-x)
Supplement: Supplementary file 1 — Supplementary Information [file 41467_2024_45501_MOESM1_ESM.pdf]

Supplementary Material: East Antarctic warming forced by ice loss during the Last Interglacial

David K. Hutchinson[1,2,*], Laurie Menviel[1,2], Katrin J. Meissner[1,3], and Andrew McC. Hogg[3,4]

[1]Climate Change Research Centre, University of New South Wales, Sydney, Australia
[2]The Australian Centre for Excellence in Antarctic Science, University of Tasmania, Hobart, Australia
[3]ARC Centre of Excellence for Climate Extremes, University of New South Wales, Sydney, Australia
[4]Research School of Earth Sciences, Australian National University, Canberra, Australia
[*]Correspondence: david.hutchinson@unsw.edu.au

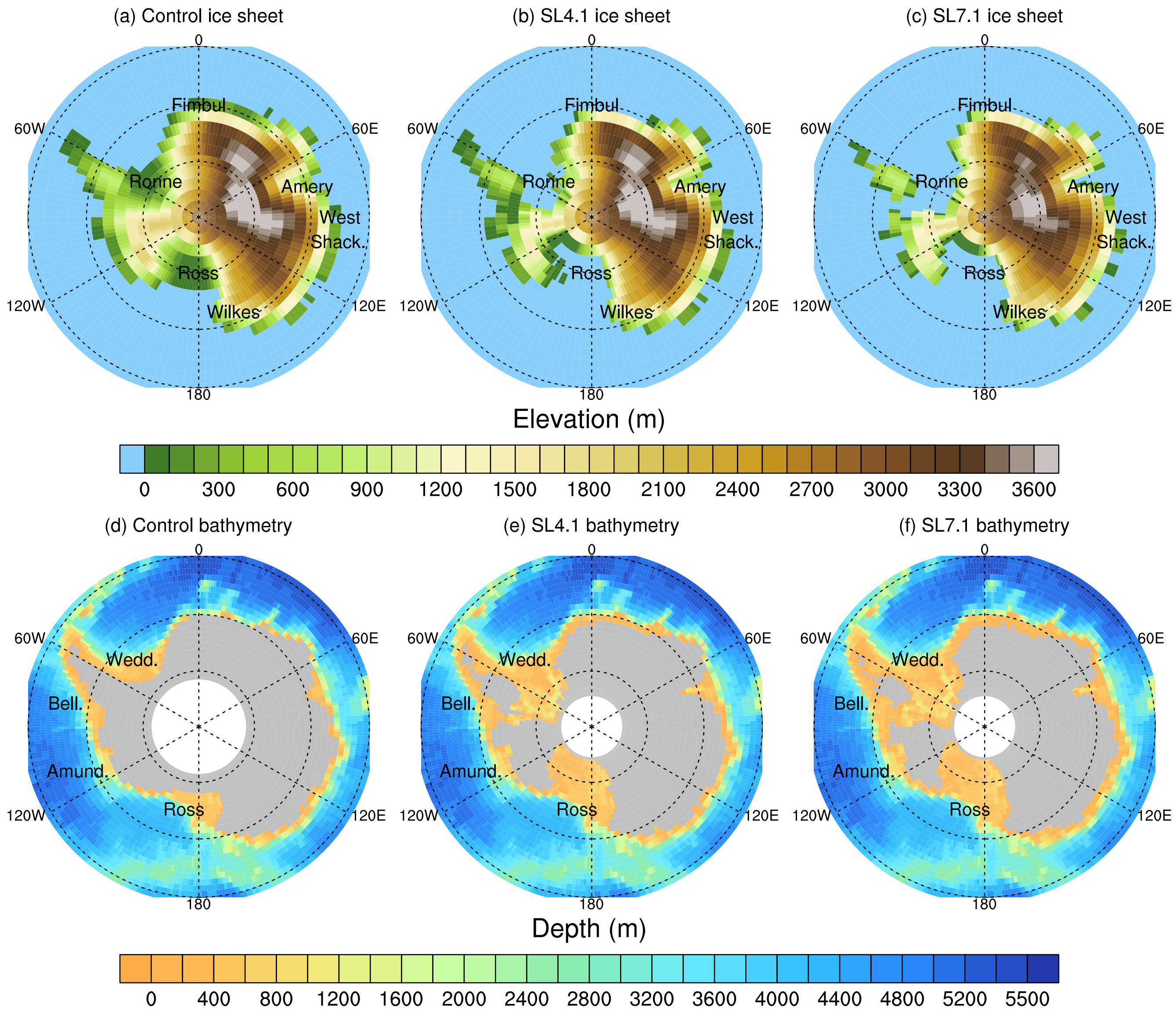

Figure S1. **Absolute values of topography and bathymetry.** Land-based topography in the (a) LIG control, (b) SL4.1 and (c) SL7.1 ice sheet configurations; bathymetry in the (d) LIG control, (e) SL4.1 and (f) SL7.1 configurations. Locations of prominent ice shelves or ice sheets are labelled in (a,b,c), while Antarctic Seas mentioned in this study are labelled in (d,e,f). Abbreviations: Shack. = Shackleton, Amund. = Amundsen, Bell. = Bellingshausen, Wedd. = Weddell.

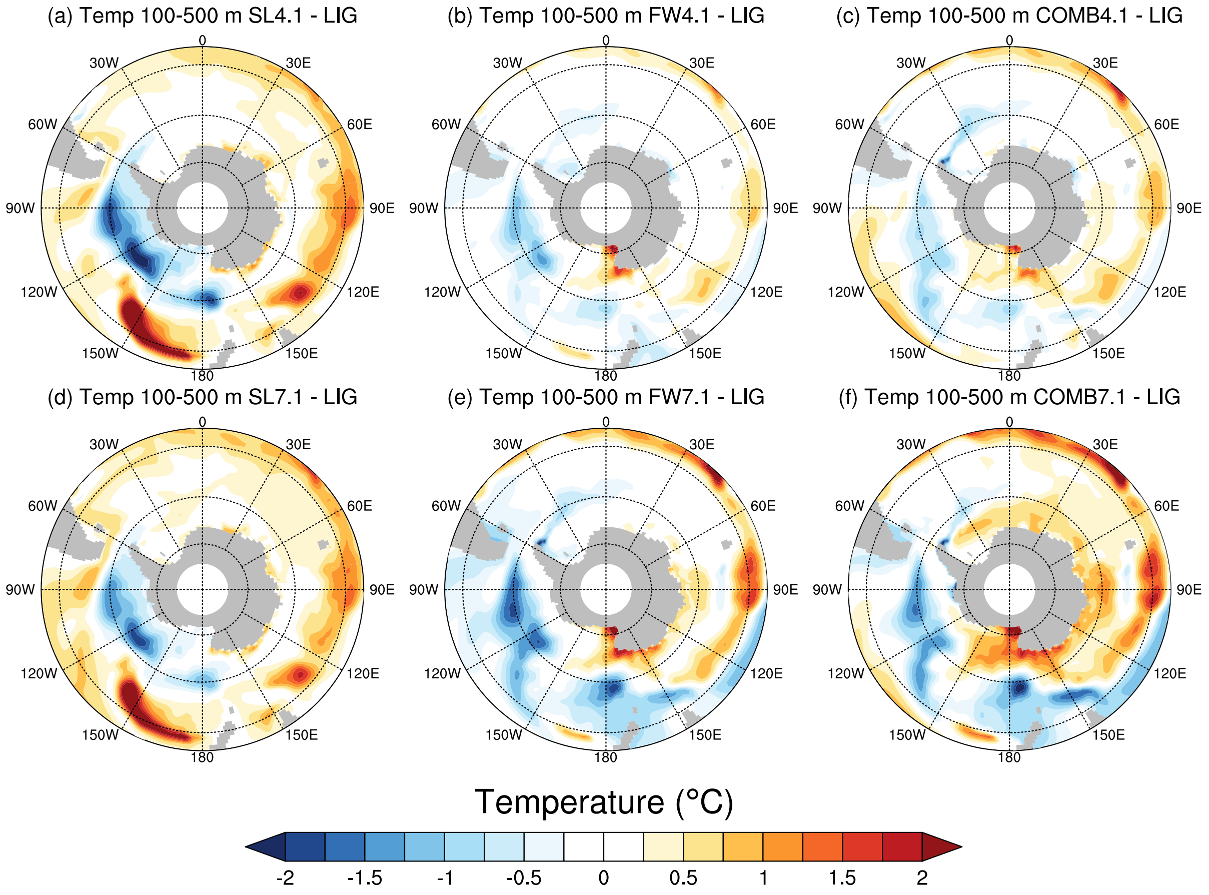

Figure S2. **Subsurface temperature anomalies, averaged over 100-500 m depth.** (a) SL4.1, (b) FW4.1, (c) COMB4.1 experiments; and (d) SL7.1, (e) FW7.1 and (f) COMB7.1 experiments compared with the LIG control.

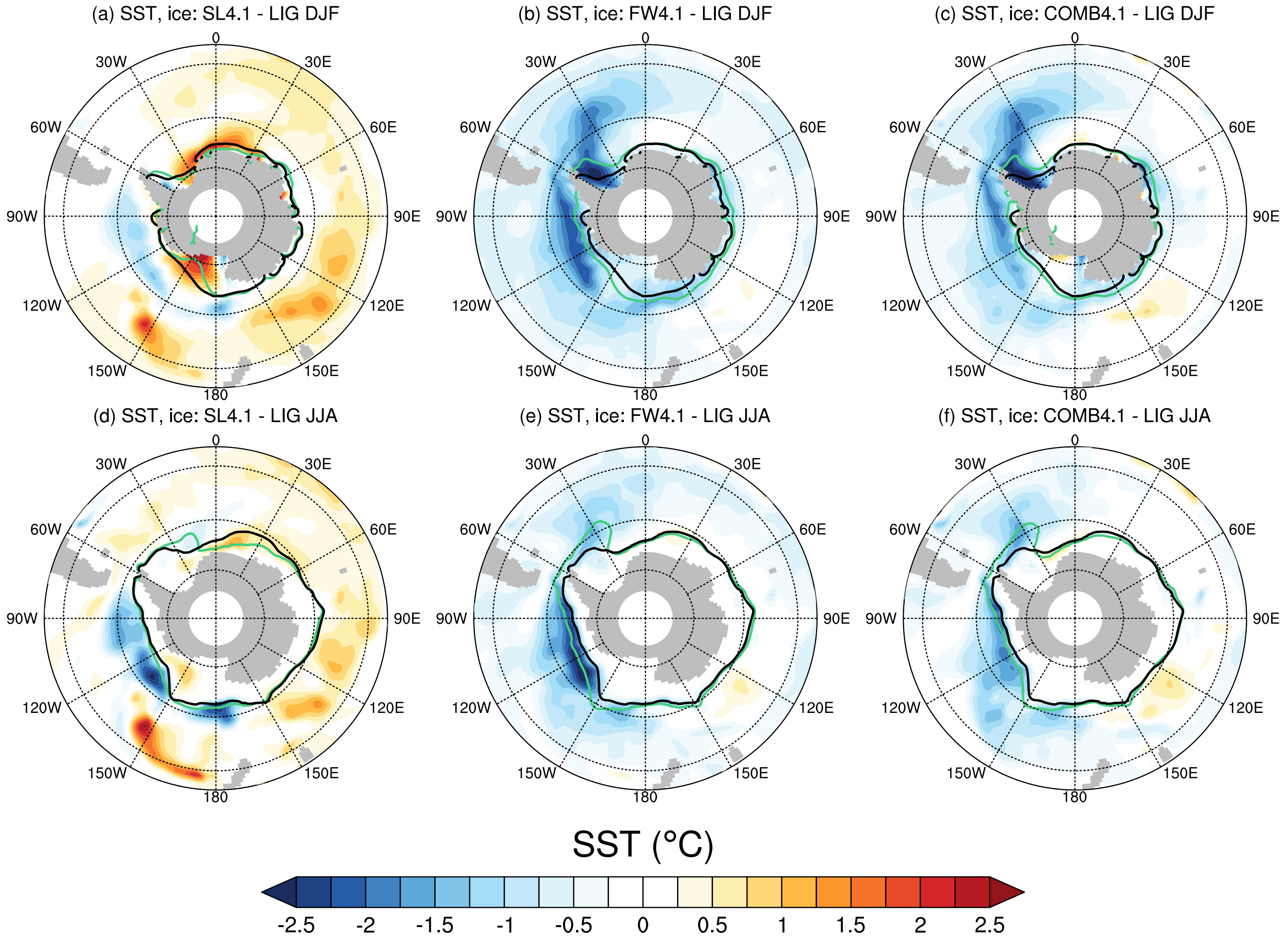

Figure S3. **Seasonal sea surface temperature (SST) anomalies with respect to the LIG simulation.** Summer (December, January, February; top row) and winter (June, July, August; bottom row), with sea ice edge shown for the LIG control (black) and perturbation experiments (green). (a,d) SL4.1, (b,e) FW4.1 and (c,f) COMB4.1 experiment.

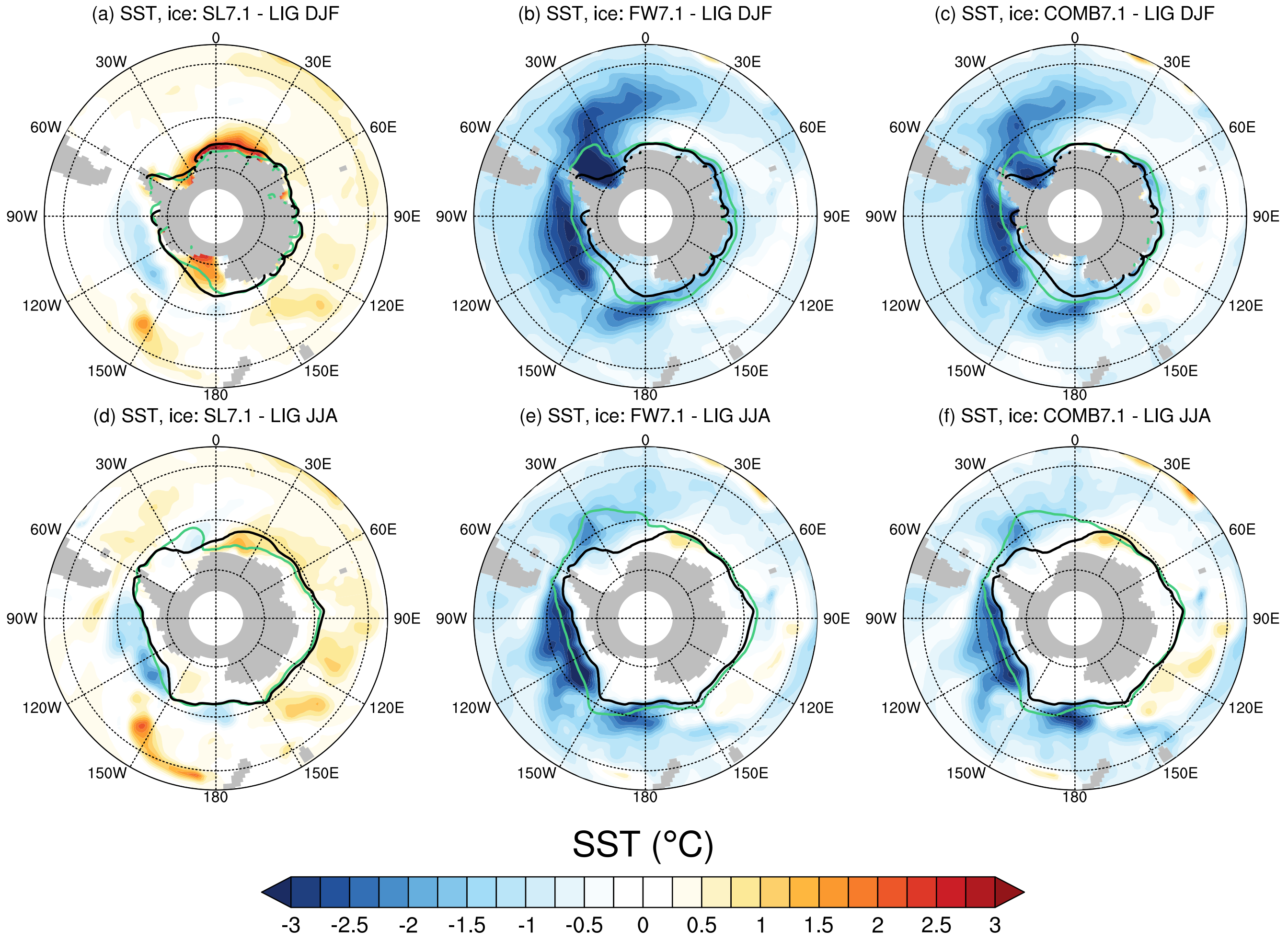

Figure S4. **Seasonal sea surface temperature (SST) anomalies with respect to the LIG simulation.** Summer (December, January, February; top row) and winter (June, July, August; bottom row), with sea ice edge shown for the LIG control (black) and perturbation experiments (green). (a,d) SL7.1, (b,e) FW7.1 and (c,f) COMB7.1 experiment.

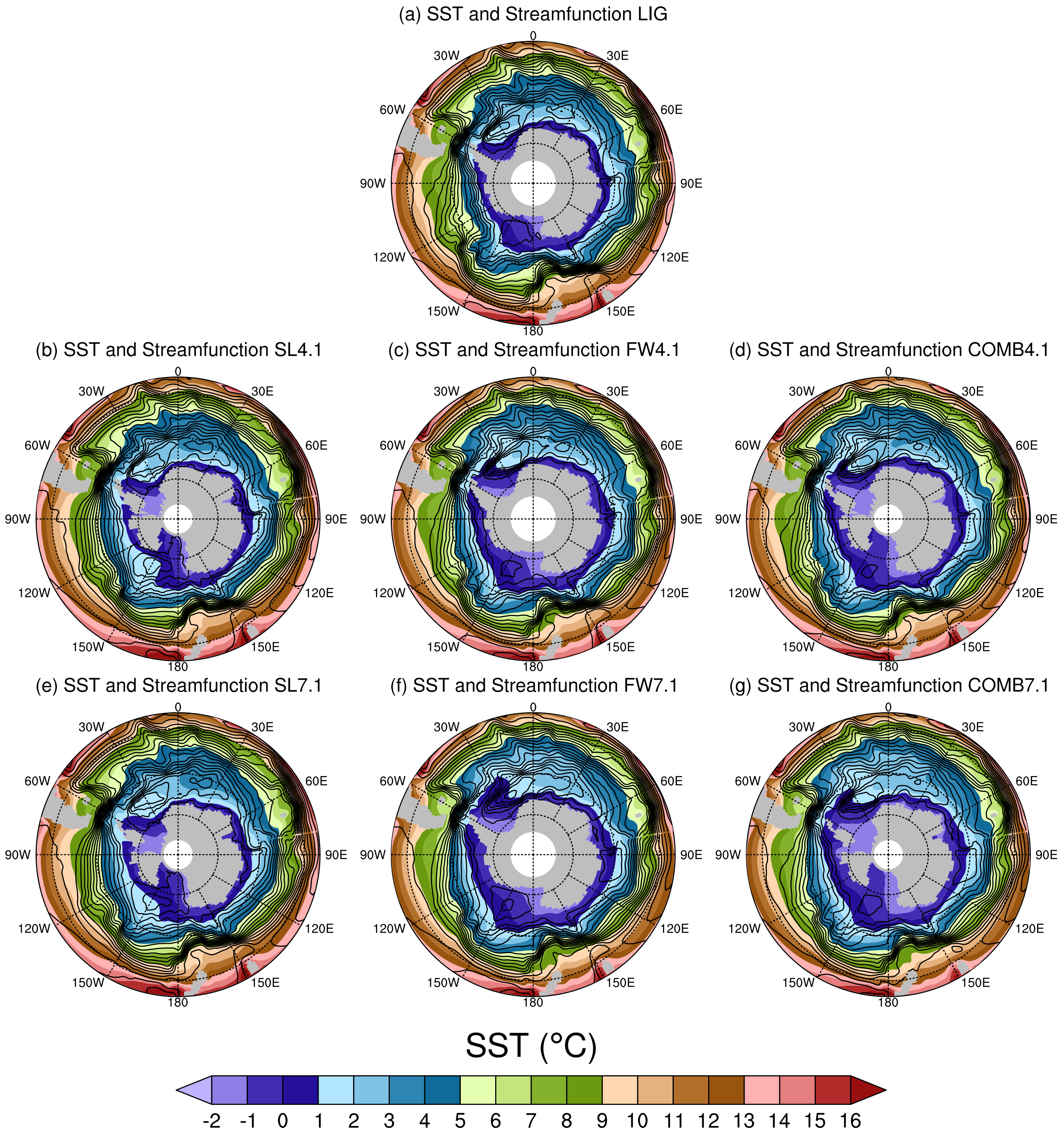

Figure S5. Absolute values of sea surface temperature (SST; colours) with barotropic streamfunction overlaid (10 Sv contour interval). Both the SST contours and streamfunction illustrate the changing pathways of the ACC. Subplots show (a) LIG control, (b) SL4.1, (c) FW4.1, (d) COMB4.1, (e) SL7.1, (f) FW7.1, (g) COMB7.1 experiments.

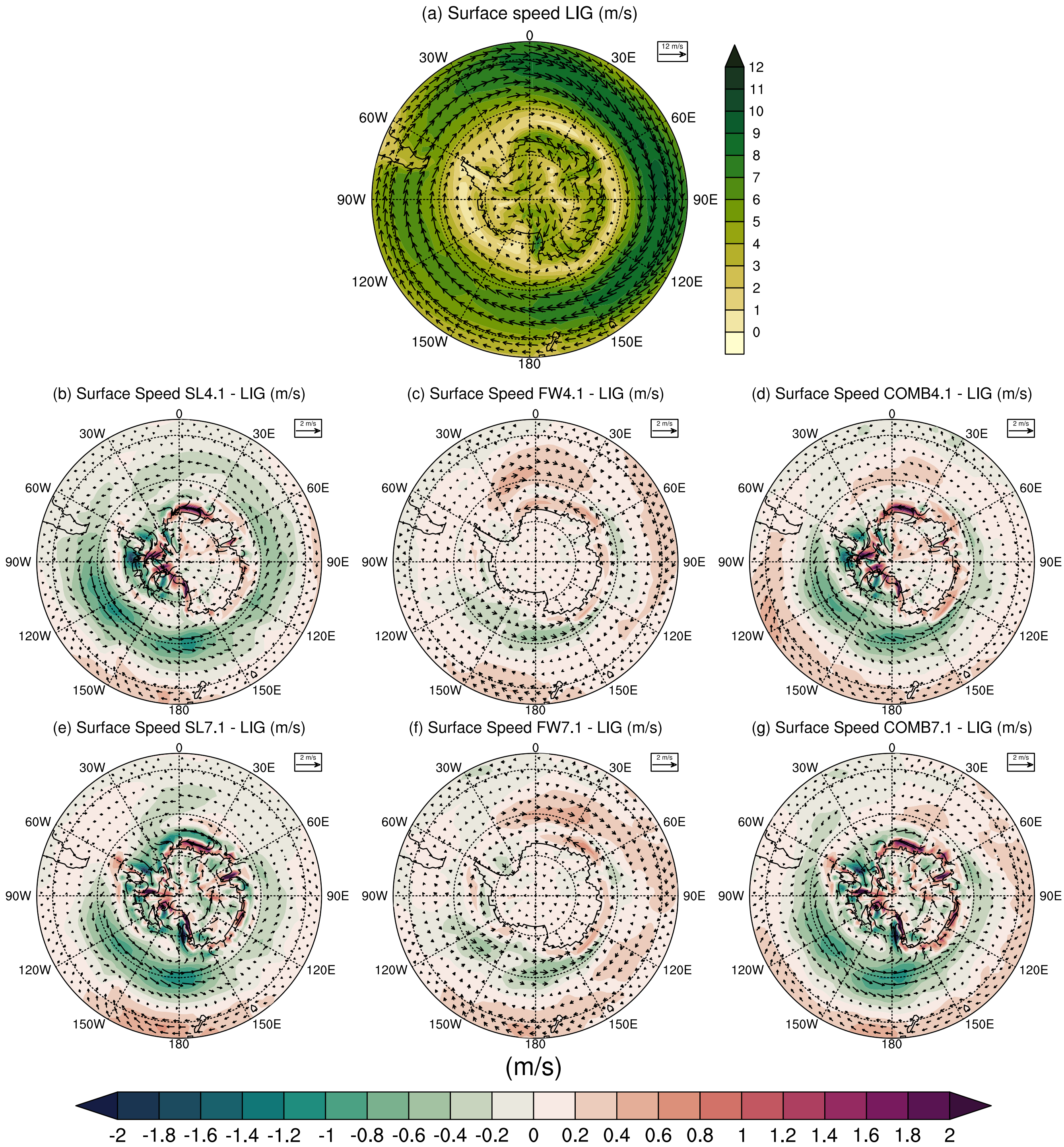

Figure S6. **Annual mean surface wind speed (at 10 m height).** (a) The LIG control experiment; and anomalies with respect to the LIG control for the (b) SL4.1, (c) FW4.1, (d) COMB4.1, (e) SL7.1, (f) FW7.1, (g) COMB7.1 experiments.

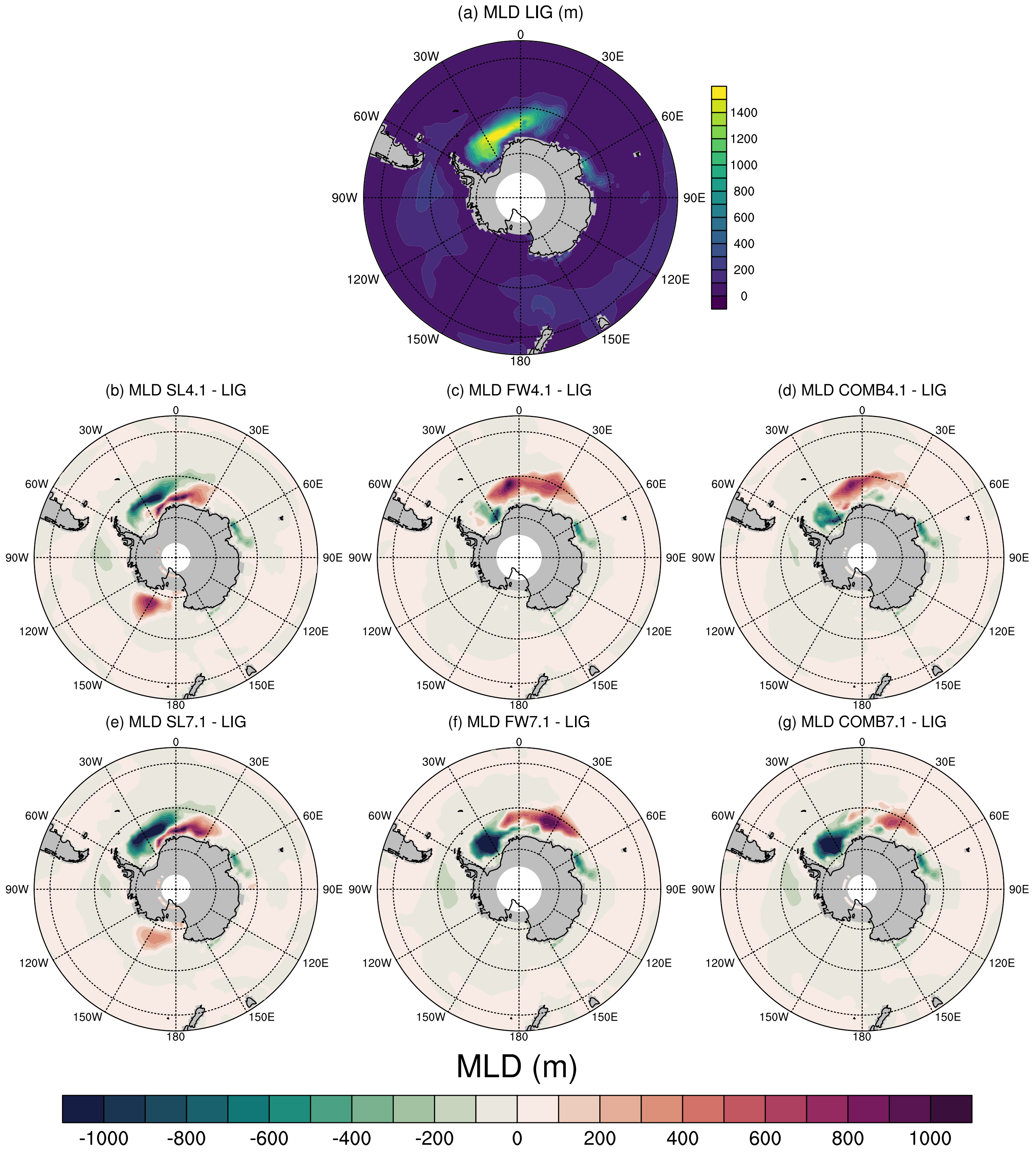

Figure S7. **Annual mean mixed layer depths (in metres).** (a) The LIG experiment; and anomalies with respect to the LIG control for the (b) SL4.1, (c) FW4.1, (d) COMB4.1, (e) SL7.1, (f) FW7.1, (g) COMB7.1 experiments.

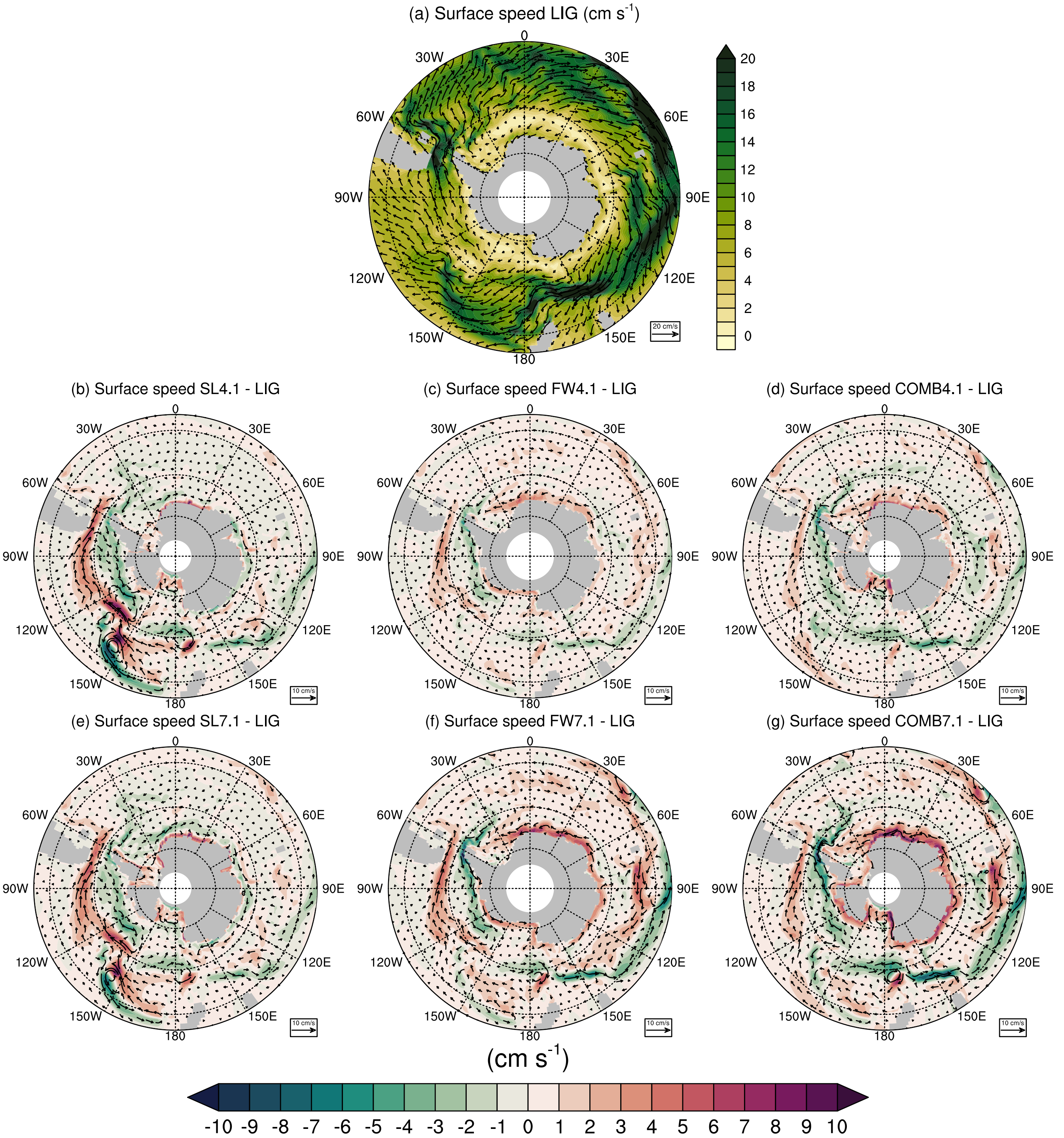

Figure S8. Ocean surface velocities. (a) The LIG control, and anomalies with respect to the LIG control for the (b) SL4.1, (c) FW4.1, (d) COMB4.1, (e) SL7.1, (f) FW7.1, (g) COMB7.1 experiments. Note that where new ocean grid cells were created, the 'anomaly' was taken with respect to a zero initial value.

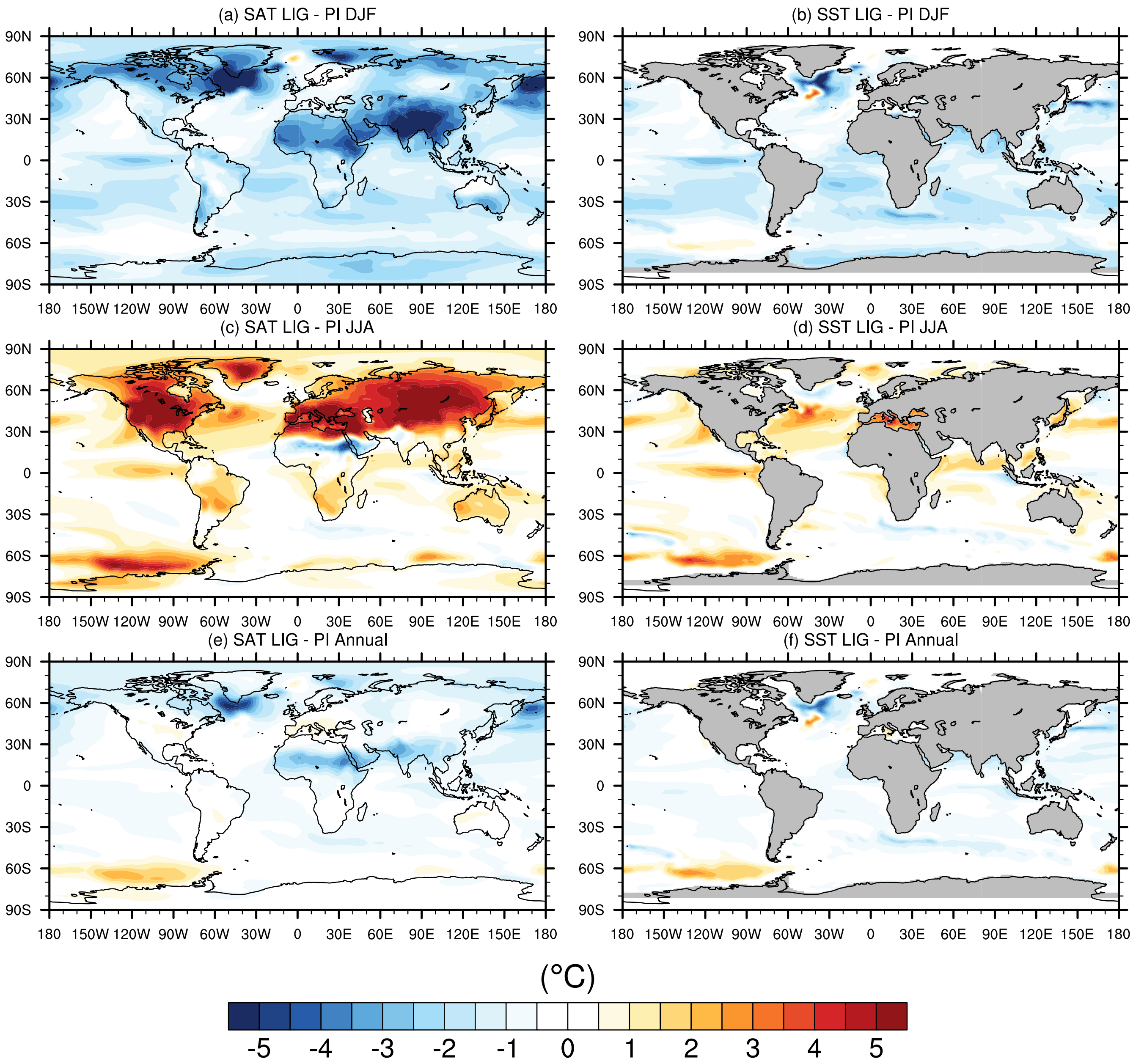

Figure S9. Summer, winter and annual temperature anomalies of the LIG minus PI experiment for surface air temperature (SAT) and sea surface temperature (SST). (a,b) December, January, February (DJF) (c,d) June, July, August (JJA) and (e,f) annual anomalies.

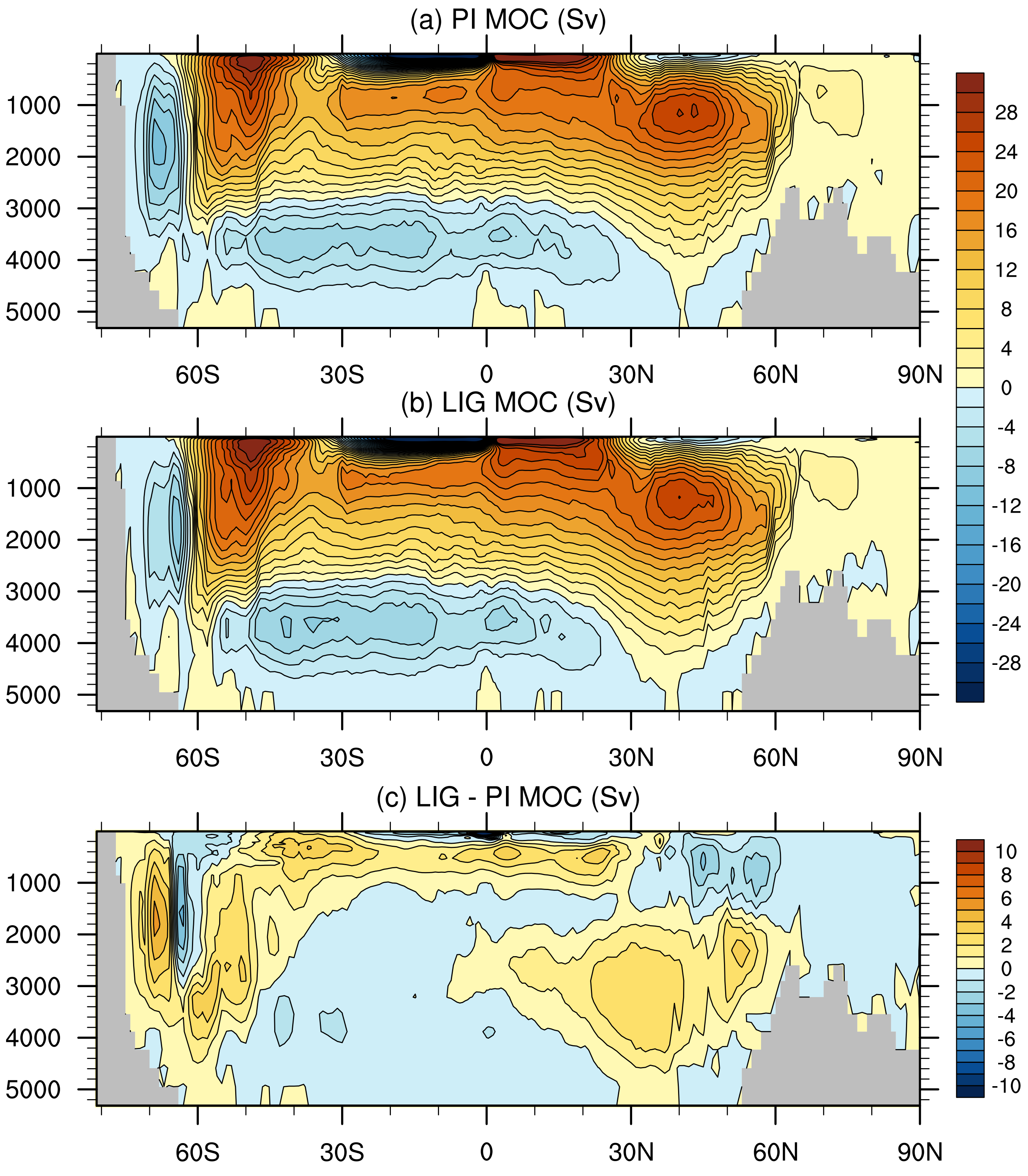

Figure S10. Meridional overturning circulation (MOC). (a) PI, (b) LIG experiments and (c) LIG - PI anomaly.

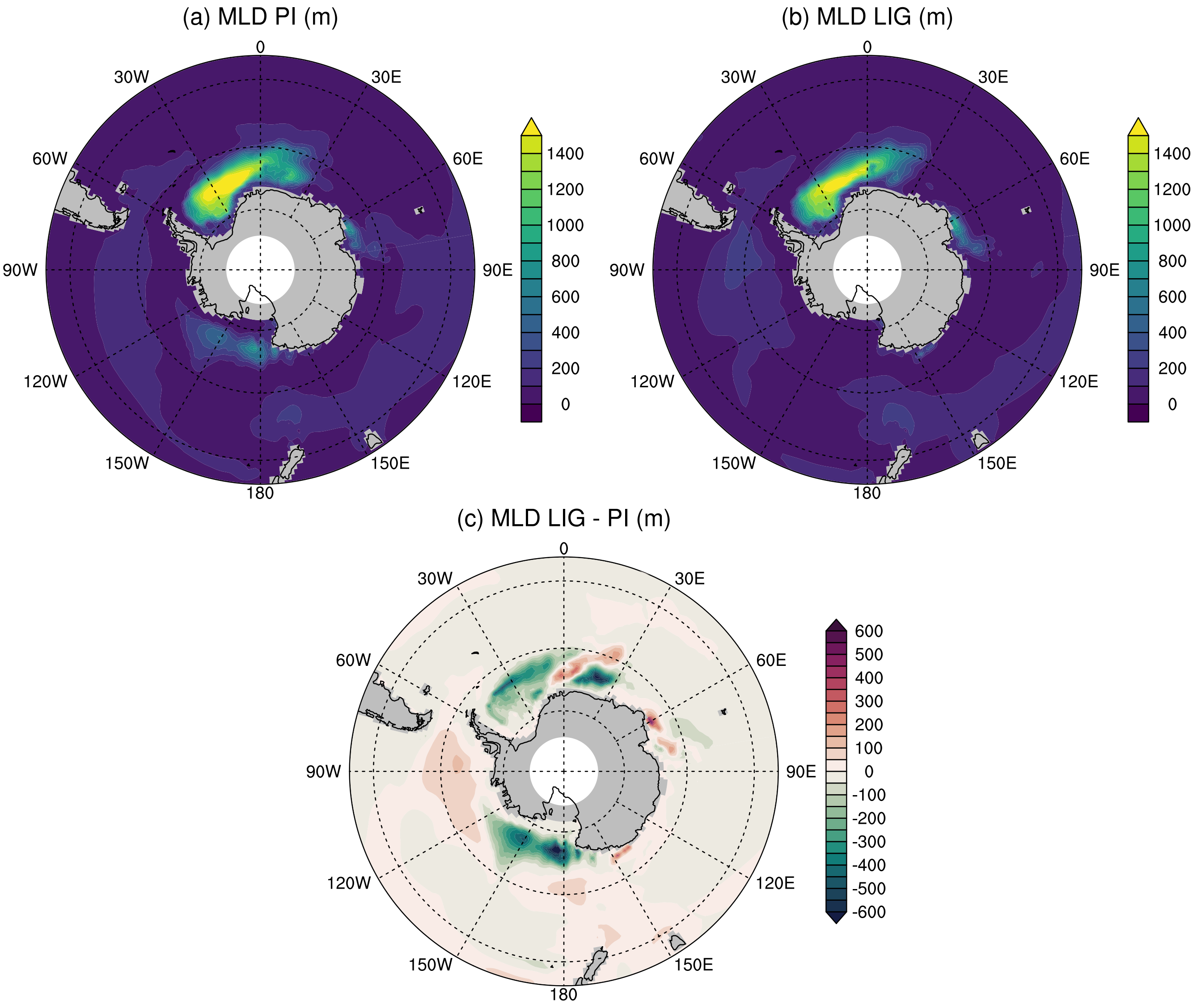

Figure S11. Mixed layer depths. (a) PI, (b) LIG experiments and (c) LIG - PI anomaly.

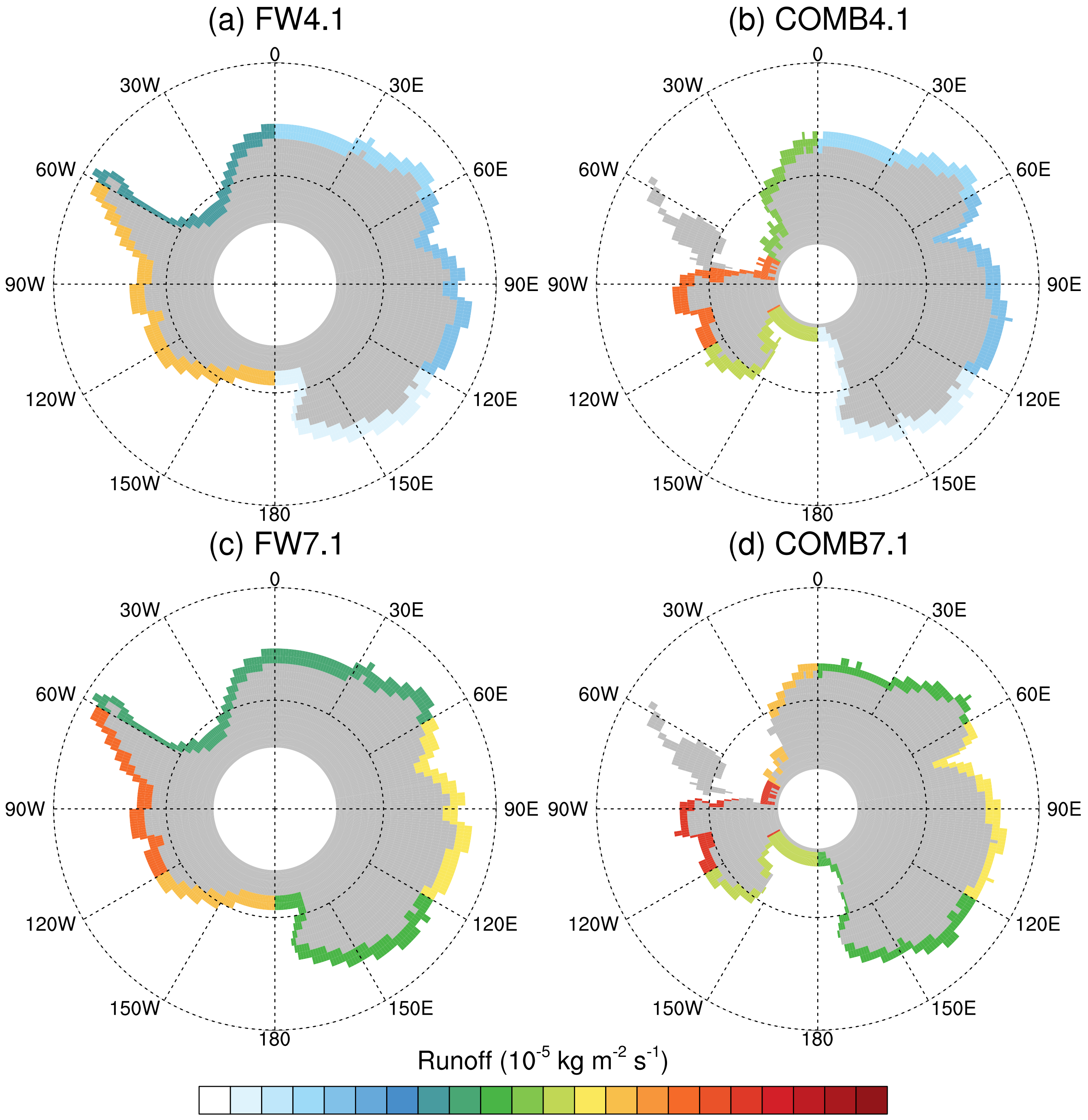

Figure S12. Distribution of meltwater runoff in each experiment. (a) FW4.1, (b) COMB4.1, (c) FW7.1 and (d) COMB7.1 experiments, using 60 ° longitudinal sectors of ice loss as shown in Figure 1a,b.
