# Supplementary material for: East Antarctic warming forced by ice loss during the Last Interglacial
Source: Nat Commun. 2024 Feb 3;15:1026. doi: 10.1038/s41467-024-45501-x (PMC10838265; doi:10.1038/s41467-024-45501-x)
Supplement: Supplementary file 3 — Description of Additional Supplementary Files [file 41467_2024_45501_MOESM3_ESM.pdf]

Description of Additional Supplementary Files:

Supplementary Data 1: Annual mean proxy-model comparison, showing sea surface temperature (-40 to -54.22 S) and surface air temperature (-75 to -79 S)

Supplementary Data 2: Summer (DJF) mean proxy vs model SST anomalies

Supplementary Data 3: Summary table of experiments. Here, `G15' refers to Golledge et al (2015), using their RCP4.5 scenario at year 5000, using their `high-melt' scenario which uses a sub-grid basal melting parameterisation. Experiments SL4.1, FW4.1, COMB4.1; SL7.1, FW7.1 and COMB7.1 were all initiated from year 1000 of the LIG experiment.
